# Prevalence of Mental Disorders and Suicidal Thoughts Among Community-Dwelling Elderly Adults 3 Years After the Niigata-Chuetsu Earthquake

**DOI:** 10.2188/jea.JE20100093

**Published:** 2011-03-05

**Authors:** Yuriko Suzuki, Atsuro Tsutsumi, Maiko Fukasawa, Hiroko Honma, Toshiyuki Someya, Yoshiharu Kim

**Affiliations:** 1 Department of Adult Mental Health, National Institute of Mental Health, National Center of Neurology and Psychiatry, Kodaira, Tokyo, Japan; 2 Niigata Institute for Traumatic Stress, Niigata, Japan; 3 Department of Psychiatry, Niigata University Graduate School of Medicine and Dental Sciences, Niigata, Japan

**Keywords:** prevalence, mental disorders, suicidality, older adults, natural disasters

## Abstract

**Background:**

Japan is located in an area prone to natural disasters, and major earthquakes have occurred recently in rural areas where the proportion of elderly adults is high. Although elderly persons are vulnerable members of communities at a time of disaster, the prevalence of mental disorders among this population has yet to be reported in Japan. This study aimed to determine the prevalence of mental disorders and suicidal thoughts among community-dwelling elderly persons 3 years after an earthquake and to identify risk factors associated with their quality of life (QOL).

**Methods:**

Face-to-face interviews were conducted with 496 community-dwelling persons aged 65 years or older in areas of Japan where 2 major earthquakes had occurred during a 3-year period. The main outcome was diagnosis of a mental disorder or suicidality.

**Results:**

During the 3-year period after the earthquake, 1.6% of men and 5.5% of women had received a diagnosis of major depression. There were no cases of posttraumatic stress disorder. Women were more likely than men to report suicidality (7.8% vs 3.8%, *P* = 0.075).

**Conclusions:**

The prevalence of mental disorders was lower than that reported in previous studies. Despite the low prevalence of mental disorders, the percentage of community-dwelling elderly persons with subclinical mental health symptoms was high. The results indicate that appropriate public health and medical interventions are warranted after a natural disaster.

## INTRODUCTION

Japan is located in an area prone to natural disasters: more than 20% of earthquakes measuring 6.0 or higher on the Richter scale occur in Japan.^1^ Since the great Hanshin-Awaji earthquake in 1995, mental health care after a natural disaster has been a concern of mental health professionals and the general public. Unlike physical care provided during a disaster, mental health care requires service provision over a longer period, in conjunction with other community recovery efforts. There has been an accumulation of research on trauma-specific reactions, such as posttraumatic stress disorder (PTSD), as manifestations of mental health problems in the aftermath of a disaster. The prevalence of such reactions is reported to range from 4.4% to 24.2%.^2^^,^^3^ However, at the time of a disaster, a broader range of mental health problems present in the community, including depression and suicidality, also warrants consideration when developing disaster recovery plans.

In Japan, major earthquakes have occurred recently in rural areas where the proportion of elderly persons is high. Although elderly adults are vulnerable members of communities at the time of a disaster, the prevalence of mental problems among this population has yet to be reported in Japan. Thus, the present study aimed to determine the prevalence of mental disorders and suicidal thoughts in the 3 years after the Niigata-Chuetsu earthquake in community-dwelling elderly persons living in a severely affected region. In addition, we investigated factors associated with subjective quality of life (QOL), including disaster-related damage and socioeconomic indicators.

## METHODS

### Study design

In this cross-sectional study, trained health professionals visited residents’ households and administered structured interviews after obtaining informed consent from the participants.

### Setting

On 23 October 2004, a major earthquake of magnitude 6.8 on the Richter scale hit central Niigata Prefecture in Japan, resulting in more than 60 deaths—mostly elderly persons and children—and 4800 injured. Damage to housing was extensive, and 103 000 residents were forced to evacuate.

Ojiya City was located near the epicenter and was officially designated a most severely affected region. We selected 3 geographic areas from this city for the sampling procedure. These areas experienced a seismic intensity of 6 or higher on the Japanese 7-stage seismic scale. More than half of the residential buildings in these areas were officially registered as half-collapsed or severely damaged. These areas were chosen as the study site because of the severity of the damage. As part of a health program, home visits were made to households in these 3 areas. During the planning of this study, another major earthquake hit the same region 3 years later, in 2007, causing several deaths, of which a disproportionately high number occurred among the elderly population. The region in this research is a rural mountainous area where the rate of elderly adults has increased as the general population has fallen.

### Participants

Among the 720 households in the 3 specific areas selected for this study, all men and women aged 65 years or older were identified using the local resident registry and approached for the survey. We targeted for interview all 900 residents living in the 3 specific areas who were on the Ojiya City registry. Among these 900 residents, 42 were dead, 20 were hospitalized, 15 were institutionalized, and 24 had moved out of the city as of October 2007. Interviewers approached the remaining 799 residents, among whom 27 were not at home, 71 had difficulty participating in the interview due to impaired hearing or vision, and 205 declined to participate in the interviews. Thus, a total of 496 community-dwelling elderly adults completed the interviews (completion rate: 62.1%).

During the first part of the interview, data regarding participant characteristics, including disaster damage information, were obtained and the presence of cognitive impairment was briefly evaluated using a digit span test. To exclude those with probable cognitive impairment, interviewees were asked to repeat 3- and 4-digit numbers backward. The interview was ended if a participant did not give informed consent during the diagnostic interview or if they gave an incorrect answer for a 3-digit span. Thus, they were not asked questions about QOL or other detailed, disaster-related information.

### Variables/measurements

*Main outcome:* Diagnosis of mental disorders—Trained health professionals administered the Japanese version of the Mini-International Neuropsychiatric Interview (MINI)^4^ and evaluated the interviewees’ mental health problems, specifically, major and minor depression, suicidal tendency, PTSD, and alcohol-related problems, both at the time of the interview and over the 3-year period since the first earthquake. The MINI was originally developed for use in clinical and research settings as a short and efficient diagnostic interview assessment of mental disorders over a 2-week, or 12-month, time frame. The suicidality module includes specific questions to assess suicidal ideation within the past month (Did you think you would be better off dead or wish you were dead? Did you want to harm yourself? Did you think about suicide?), suicide plan within the past month (Did you have a suicide plan?), suicide attempt within the past month (Did you attempt suicide?), and lifetime suicide attempts (In your lifetime, did you ever make a suicide attempt?). Current suicide risk is based on a weighted point system that classifies the risk as low, medium, or high.

Before the interview, the health professionals underwent a training session for the diagnostic interview. The 18 interviews were audiotaped and simultaneously assessed by 5 raters. To evaluate interrater reliability, the intraclass correlation coefficients (ICC) for total counts of symptoms for 3 different diagnoses (major depressive episode, PTSD, suicidality) were calculated. The rating for the 3 diagnoses was reliable, with a range of 0.889 to 1.000. We modified the interview so that people with minor depression were defined as those who did not meet the criteria for a major depressive episode, but nevertheless had experienced an episode of depressive mood or lack of interest/pleasure and had a total of 2 to 4 items on the depression module.

*Secondary outcome:* Subjective quality of life—we administered the Japanese version of the World Health Organization Quality of Life-BREF (WHOQOL-BREF),^5^ a self-report questionnaire, and interviewers read the text out loud or clarified the meaning, as needed. On administering this questionnaire, the item on sexual activity was not included, as it was deemed culturally inappropriate for elderly adults living in rural areas of Japan. The overall score, as well as the subscores for the physical, psychological, social, and environmental subdomains, were calculated according to the scoring procedure specified in the manual, except for changing the total number of items to 25.^5^^–^^7^

*Predictors:* Questions were asked to elicit data on a number of variables: (1) present socioeconomic factors, including gender, age, years of education, and marital status; (2) disaster-related factors; (3) personal vulnerability, including current physical illnesses, and lifetime history of psychiatric visits; and (4) post-disaster factors, including number of cohabitants.

### Ethical considerations

Before the interviews, the aims and procedures of the study were explained clearly and simply by the interviewers using written materials. Verbal consent was obtained from the participants due to cultural distrust of written consent. The process of obtaining consent was documented by the interviewers. All procedures in the present study were approved by the Ethics Committee of the National Center of Neurology and Psychiatry, Japan.

### Statistical analysis

We described the participants’ characteristics and then determined the 2-week point and 3-year period prevalences of mental disorders by gender. To identify factors associated with mental health problems, ie, current major depressive disorder, minor depressive disorder, PTSD, alcohol dependence/abuse, and suicidality, the distributions of basic characteristics and QOL subscore were compared between participants with and without mental health problems. Then, multiple regression analysis was performed to identify the factors associated with poor QOL. Based on previous research on disaster mental health, the variables selected for the model were socioeconomic factors (gender, age, marital status, and years in education), disaster-related factors (severity of the disaster damage), individual vulnerability (history of psychiatric visits, and current physical illnesses), and post-disaster support (number of cohabitants). Furthermore, multicollinearity between variables was evaluated, but not found. The number of subjects included in the statistical analysis varied due to missing data for some variables. The statistical analysis and 95% confidence intervals (CIs) presented here were generated using Stata Ver10 (Collage Station, TX, USA). To control for Type I error inflation, a 2-tailed *P* value of less than 0.05 was considered significant.

## RESULTS

The demographic characteristics of the participants and nonparticipants are presented in Table 1. Data on the severity of disaster damage in 2004 were not available from 20.8% of nonparticipants versus 1.4% of participants. There were no other significant differences between interview participants and nonparticipants. A total of 52 (10.5%) participants were unable to repeat a 3-digit number backward and were thus excluded from the latter part of the diagnostic interview.

**Table 1.** Characteristics of study participants 3 years after the 2004 Niigata-Chuetsu earthquake

	Interview participants (*n* = 496)		Nonparticipants (*n* = 303)	
		
	*n*/median	%/IQR^a^	*n*/median	%/IQR^a^
Gender				
Male	205	41.3	136	44.9
Female	291	58.7	167	55.1
Age, years				
65–74	219	44.2	132	43.6
75+	277	55.9	171	56.4
median/IQR^a^	76	10.5	75	10.0
Marital status				
Married	345	69.6		
Divorced	3	0.6		
Bereaved	146	29.4		
Never married	2	0.4		
Years of education				
median/IQR^a^	8	3		
Living alone				
yes	33	6.7	13	4.3
no	462	93.3	290	95.7
Number of cohabitants				
median/IQR^a^	4	3	4	4
Disaster damage in 2004				
Total collapse	47	9.6	26	8.6^b^
Massive collapse	41	8.4	21	6.9
Half collapse	185	37.7	102	33.7
Partial collapse	199	40.5	86	28.4
None	12	2.4	5	1.7
Unknown	7	1.4	63	20.8
Disaster damage in 2007				
Half collapse	1	0.2		
Partial collapse	16	3.2		
None	471	95.3		
Unknown	6	1.2		
Lifetime psychiatric visits	20	4.3		
Digit span test (3 digits)				
Incorrect	52	10.5		

Chi-square test or *t*-test was used. ^a^Interquartile range, ^b^*P* < 0.01.

The point prevalence of major depression 3 years after the Niigata-Chuetsu earthquake was 0.5% in men and 0.4% in women, and the respective values for minor depression were 1.6% and 1.2%. Although most participants (98.7%, *n* = 442) experienced the earthquake, no participant met the criteria for a diagnosis of PTSD 3 years after the event. Experience of alcohol problems was reported only by men: the point prevalence was 3.9% for alcohol dependence and 2.2% for alcohol misuse. Three years after the event, 3.2% of men and 6.6% of women reported varying degrees of suicidality. It was noteworthy that 18 of the 23 people who reported suicidality (78.3%) for the 2-week period and 15 of the 27 people who reported it for the 3-year period (55.6%) were assessed as having a low level of suicidality.

During the 3 years after the earthquake, major depression was diagnosed in 1.6% of men and 5.5% of women, a significant difference (*P* = 0.045); minor depression was diagnosed in 2.8% of men and 4.5% of women, and the difference was not significant. More women than men reported suicidality, but the difference was not significant (7.8% vs 3.8%, *P* = 0.107; Table 2).

**Table 2.** Prevalence of depression, PTSD, alcohol abuse, and suicidality in the previous 2 weeks and previous 3 years among community-dwelling elderly males and females

Mental disorder	All		Male		Female		*P*-value
		
	*n*	%	*n*	%	*n*	%	
Major depressive disorder							
2 weeks ​ (*n* = 443)	2	0.5	1	0.5	1	0.4	1.000
3 years ​ (*n* = 442)	17	3.9	3	1.6	14	5.5^a^	0.045
Minor depressive disorder							
2 weeks ​ (*n* = 443)	6	1.4	3	1.6	3	1.2	0.697
3 years ​ (*n* = 425)	16	3.8	5	2.8	11	4.5	0.443
PTSD (current)							
Earthquake ​ (*n* = 442)	0	0.0	0	0.0	0	0.0	
Other events ​ (*n* = 441)	0	0.0	0	0.0	0	0.0	
Alcohol- (current)							
Dependence ​ (*n* = 440)	7	1.6	7	3.9	0	0.0^b^	0.002
Abuse ​ (*n* = 441)	4	0.9	4	2.2	0	0.0^a^	0.029
Suicidality							
2 weeks ​ (*n* = 443)	23	5.2	6	3.2	17	6.6	0.133
3 years ​ (*n* = 441)	27	6.1	7	3.8	20	7.8	0.107

All tested using Fisher’s exact test. ^a^*P* < 0.05, ^b^*P* < 0.01.

Those with and without current mental health problems were compared in terms of basic characteristics, disaster-related information, and quality of life (Table 3). Only lifetime psychiatric visits was disproportionately reported among those with current mental health problems. In the physical and psychological domains, QOL subscores were lower in those with current mental health problems than in those without such problems.

**Table 3.** Characteristics of participants with and without current mental health problems^a^

	Mental health problem			
	
	Absent		Present	
	(*n* = 408)	%	(*n* = 36)	%
Gender				
Male	167	40.9	18	50.0^b^
Female	241	59.1	18	50.0
Age, years				
65–74	192	47.1	13	36.1^b^
75+	216	52.9	23	63.9
Marital status				
Married	287	70.3	23	63.9^b^
Divorced	3	0.7	0	0.0
Bereaved	116	28.4	13	36.1
Never married	2	0.5	0	0.0
Living alone				
Yes	28	6.9	1	2.8^b^
No	379	93.1	35	97.2
Disaster damage in 2004				
Total collapse	40	9.9	5	13.9^b^
Massive collapse	29	7.2	2	5.6
Half collapse	153	37.9	14	38.9
Partial collapse	163	40.4	15	41.7
None	12	3.0	0	0.0
Unknown	7	1.7	0	0.0
Disaster damage in 2007				
Half collapse	1	0.3	0	0.0^b^
Partial collapse	11	2.7	2	5.7
None	390	95.8	32	91.4
Unknown	5	1.2	1	2.9
Lifetime psychiatric visits				
Yes	14	3.7	4	12.5^b,e^
No	369	96.3	28	87.5

	mean	sd	mean	sd
	
Quality of life				
Physical domain	3.69	0.55	3.37	0.69^c,f^
Psychological domain	3.48	0.49	3.28	0.58^d,e^
Social domain	3.81	0.56	3.70	0.68^d^
Environmental	3.40	0.59	3.46	0.82^c^
Overall	3.53	0.40	3.35	0.52^c^

^a^Mental health problems comprised current major depressive disorder, minor depressive disorder, alcohol dependence/abuse, and suicidality.

^b^Chi-square test, ^c^Mann-Whitney test, ^d^*t*-test. ^e^*P* < 0.05, ^f^*P* < 0.01.

Finally, we examined risk factors for poor QOL, as evaluated by the WHOQOL-BREF (Table 4). Factors associated with poor QOL were having a physical illness (physical domain), having a physical illness and fewer cohabitants (psychological domain), and having fewer cohabitants (environmental domain). Generally speaking, in predicting overall QOL scores from the model, having more cohabitants was protective, while greater severity of disaster damage in 2004 and having a physical illness were risks for lower QOL 3 years after the earthquake.

**Table 4.** Results of regression analysis of quality of life and basic characteristics of interviewees: Standardized regression coefficients, beta

Variables	Physical *n* = 438	Psychological *n* = 437	Social *n* = 437	Environmental *n* = 436	Overall *n* = 437
Gender	−0.04	−0.07	0.06	−0.11^a^	−0.06
Age	−0.12	0.00	−0.04	−0.02	−0.04
Marital status	0.02	0.01	−0.07	−0.04	0.00
Number of cohabitants	0.06	0.10^a^	0.08	0.11^a^	0.11^a^
Years of education	−0.09	−0.01	−0.01	−0.02	−0.05
Lifetime psychiatric visits	−0.04	−0.01	−0.02	0.02	−0.01
Severity of disaster damage in 2004	−0.09	−0.06	−0.08	−0.08	−0.09^a^
Severity of disaster damage in 2007	0.03	0.00	0.06	0.07	0.04
Any physical illness	−0.20^b^	−0.13^b^	−0.01	0.01	−0.14^b^

Gender: male = 0, female = 1; Age: continuous; Marital status: divorced, bereaved, never married = 0, married = 1; Lifetime psychiatric visit: never = 0, yes = 1; Severity of disaster damage in 2004 and 2007: none = 0, partial collapse = 1, half collapse = 2, massive collapse = 3, total collapse = 4; Any physical illness: none = 0, yes = 1; ^a^*P* < 0.05, ^b^*P* < 0.01.

## DISCUSSION

We sought to determine the prevalence of mental problems and QOL issues 3 years after the Niigata-Chuetsu earthquake among community-dwelling elderly persons. Although the rate of participants with severe mental disorders was low, the rate of those with poor mental health, including episodes of major and minor depression and suicidality, was not negligible. We also sought to identify the factors associated with poor QOL 3 years after the initial 2004 earthquake and shortly after the second earthquake in 2007. Having fewer cohabitants, having more severe disaster damage in 2004, and having a physical illness were associated with poor overall QOL. These findings imply that it is necessary to plan for provision of community mental health services after a disaster, and that services that emphasize traumatic reactions may not meet the mental health needs of a specific community.

Previous studies of the point prevalence of major depression and PTSD after a disaster reported a range of 6.4% to 11%,^3^^,^^8^ which is far higher than that observed in the present study. In non-disaster settings, the prevalence of depression among community-dwelling persons aged 65 or older was found to be approximately 0.4% to 0.5% in Japan^9^^,^^10^ and 0.9% to 9.4% in Western countries.^11^ The point prevalence in the present study is thus comparable to those noted in non-disaster settings in previous research. One study with a nationally representative sample, the World Mental Health-Japan study, reported a 1-year prevalence of 2.9% for major depression, 3.1% for any type of mood disorder, and 1.6% for alcohol dependence or abuse in the adult poulation.^12^ In the same study, the 1-year prevalence was 0.8% for major depression and 0.3% for minor depression among adults aged 65 years or older.^13^ Considering the comparable point prevalences and higher period prevalence, depressive episodes were more frequent after a disaster; however, additional study of causality in disasters is necessary. It is difficult to directly compare rates between studies, due to the different target populations and durations of observation. Nonetheless, the far lower point prevalence for these mood disorders in the present study suggests that elderly persons who survive a natural disaster are resilient and that they receive valuable support from fairly well-organized health and other services. However, the higher prevalence of alcohol-related problems among men warrants further attention, especially from a cultural perspective.

Interestingly, we found no cases of PTSD in this study. In previous disaster research, PTSD was often reported, although the rate varied widely, from 4.4% to 24.2%,^2^^,^^3^ with natural disasters typically resulting in lower rates than man-made disasters. Some experts argue that while natural disasters undoubtedly negatively impact people’s lives, the psychological impact of such a calamity is less severe than that following a man-made disaster that is an expression of vicious destructive intent.^14^ In fact, the prevalence of PTSD during a 1-year period was 0.4% in a non-disaster setting in Japan,^12^ a rate that is similar to that of the present study, even though the sampled target population and the observation period were different. Elderly adults may have reacted to the event more calmly because of maturity gained from previous traumatic episodes, and because of the many other coping strategies learned throughout their lives.^15^ Indeed, some of the participants in the present study reported that their experiences during World War II were far harsher than a single earthquake. It is also possible that those most severely affected by the earthquake could not return to the area from evacuation areas because housing damage was too severe and were instead rebuilding their lives while in temporary housing or had moved to other areas with the support of others. Kato et al investigated the frequency of PTSD among adults living in temporary housing or in housing provided for those severely affected 45 to 47 months after the Hanshin-Awaji earthquake and found a point prevalence of 9.3% and a lifetime prevalence of 21.3%.^16^ Thus, in the present study, we cannot exclude the possibility that we reached only those who had satisfactorily recovered after the earthquake, and that people with severe mental health problems, including PTSD, may have declined the interview. We must therefore interpret our results with caution so as not to undervalue the effects of this traumatic event.

When we focused on cases of minor depression and suicidal tendency, which do not meet any conventional diagnostic criteria, most with suicidality were assessed as low risk on MINI, and none satisfied the diagnostic criteria for a mental disorder. The point prevalence was 1.4% for minor depression and 5.2% for suicidality in both sexes, when both minor depression and suicidality were considered 3 years after the earthquake. The point prevalence for suicidal tendency was 5.2%, which is higher than in previous studies of community-dwelling elderly persons in urban areas (4.5%) and rural areas (2.3%) of Japan.^17^^,^^18^ In our study, the response items for suicidality covered a wide range of intention, from feeling that one would rather be dead to actual suicide attempts, which may have resulted in the higher reported rate of suicidal tendency in this study. However, post-disaster subclinical mental health symptoms, including minor depression and suicidal tendency, warrant attention from public health specialists. During planning and post-disaster provision of mental health services, we must keep in mind that such services should be developed from both public health and medical models to treat specific mental disorders.

Overall, the QOL score among participants was higher than those noted in a previous study conducted in a non-disaster setting in Japan^19^ and another study of disaster in Asian countries.^2^^,^^20^ In our study, QOL score was positively predicted by number of cohabitants and negatively predicted by the severity of the disaster damage experienced and having a physical illness, which is consistent with previous findings.^8^^,^^21^^,^^22^

Based on the regression model, the physical QOL subdomain was explained by having a physical illness, which is self-explanatory. The items regarding physical QOL asked about energy level, fatigue, and activities of daily living. Thus, those who lived through severe disaster damage may also have experienced chronic additional life stress, resulting in negative effects in the physical domain. In the psychological domain, having more cohabitants had a positive effect on QOL, while having a physical illness negatively affected QOL. Overall positive thinking and negative feelings were included in this domain, thus frailty due to physical illness may have also negatively contributed to the psychological aspects of QOL. The mean social QOL score was highest among all the domains, and we found no factors associated with this domain. However, this finding must be interpreted with caution, as this domain comprised only 2 items, and its distribution was skewed. In the environmental domain, being female was a risk factor for poor QOL. This domain included items such as financial resources, transportation, and opportunities for acquiring new information and skills. In general, women have fewer opportunities to access information, services, and financial resources,^23^ and have fewer cohabitants. Therefore, QOL was lower among women in our sample. These findings are consistent with other studies; however, it is noteworthy that the model-adjusted *R*^2^s were somewhat low, which highlights the need for careful interpretation of these risk factors.

This study has several limitations. First, the diagnostic interview was conducted using the MINI, which was designed to detect individuals with mental disorders in clinical settings and asks about symptoms during a 2-week time frame. We extended the application of the MINI to investigate 3-year period prevalence after the earthquake. Although we set a specific time frame, the validity of period prevalence estimates has not been confirmed. In a community survey, the point prevalence of mental disorders is low, thus further research using a more comprehensive interview to detect a certain period prevalence or lifetime prevalence is required.

Secondly, this study focused on community-dwelling elderly persons. The prevalence of mental disorders was lower than in previous studies; however, with respect to the needs of mental health care provision after a disaster, the frailer members of the at-risk population were presumably hospitalized, institutionalized, or moved out of the area. Consequently, rates of certain subclinical mental problems, such as minor depression and suicidal ideation and tendencies, were explored in the present study, and the results indicate that appropriate public health countermeasures are warranted. Thus, we conclude that the need for mental health services should not be undervalued and that the strategies required for high-risk populations and those living in the community are different.
